# Another Method of Perforating the Drum Membrane

**Published:** 1875-07

**Authors:** Joseph Simrock

**Affiliations:** New York


### ANOTHER METHOD OF PERFORATING THE DRUM MEMBRANE.

BY JCSEPH SIMROCK, M. D., NEW YORK.

In a number of cases of chronic otitis catarrhalis media, with little or no opacity of the membrana tympani, where the Eustachian tube was previous, and the ordinary methods of treatment had been tried unsuccessfully, I resorted, instead of puncturing the membrana tympani, to a novel procedure of opening the tympanic cavity. This method consists in the application of sulphuric acid upon a selected spot of the surface of the membrana tympani, mostly on its posterior half. Such a proceeding at first sight appears hazardous, but in reality the action of this substance is wholly under control, the quantity requisite to produce a large opening being quite a minimal one. The way of applying it is to bring the end of a fine probe which has been covered with a thin layer of cotton and afterwards dipped into strong sulphuric acid, in contact with the surface of the membrane, either to a particular spot with a slight pressure, or by rubbing it over a larger field. In the first case an opening may be produced almost instantaneously; in the latter the fluid dries in, and, after awhile, the part over which it has been spread, having become anæsthetic, may be removed by a probe like so much paper tissue. There is hardly any pain caused by the operation, and in a good many cases it might be done without the patient being aware of any operative proceeding taking place at all. The openings are of course of different size, according to the more or less limited application of the acid, but they are invariably of long persistence. It is seldom that an aperture by puncture, even of large size, does not close within two or three days. Of seventeen orifices produced by the above method, three remained open for four months; the longest time the opening took to close was twelve weeks, the shortest thirty-one days. After closure it can be easily reopened by the same treatment, and by the result of several perforations in the same individuals, I am inclined to the belief, that by repeating the operation the regenerative power of the membrane is greatly diminished, perhaps entirely lost. In one patient the second perforation still persists in both ears since three and a half months; in another twelve weeks; in a third, ten weeks, without visible inclination to close again. Only in three cases I have seen slight inflammation of middle and external ear follow the operation, without any disagreeable complication however. It is too soon to compare the results of puncture by the needle or of tenotomy of the tensor tympani, but I am of opinion that they will compare favorably with either. In seventeen cases the tinnitus disappeared in five; in nine it was so much diminished that patients were no longer troubled; three remained unimproved. The improvement for hearing, especially for conversation, was very manifest in six; less so in four; no improvement for hearing in seven. This procedure may also be useful in a diagonistic view; orifices made intentionally so large as to occupy one-half to three-quarters of the whole membrane enabled me in various cases to see pathological changes within the tympanic cavity: small osseous growths on the promontory membranous band in different places, in two cases anæsthesia of the promontory, on pressure; in one case I found the entrance of the fenestra rotunda filled up with osseous matter.

After an opening has been established, syringing of the ear must be avoided, even if a slight discharge should set in.—*New York Medical Record.*

---

*Anti-Emetic Properties of Tobacco.*—A young girl, not pregnant, suffering from severe and unchecked vomiting, under Dr. Beaumetz's care, at the Hotel Dieu, was, after ineffectual trial of various remedies, ordered to smoke a cigarette after each meal. This, so long as its use was persisted in, seemed to control the vomiting entirely.—*New Remedies.* J. B. M.

